# The optimal use of tildrakizumab in the elderly via improvement of Treg function and its preventive effect of psoriatic arthritis

**DOI:** 10.3389/fimmu.2023.1286251

**Published:** 2023-10-19

**Authors:** Takemichi Fukasawa, Takashi Yamashita, Atsushi Enomoto, Yuta Norimatsu, Satoshi Toyama, Asako Yoshizaki-Ogawa, Shoko Tateishi, Hiroko Kanda, Kiyoshi Miyagawa, Shinichi Sato, Ayumi Yoshizaki

**Affiliations:** ^1^ Department of Dermatology, Psoriasis Center, The University of Tokyo Graduate School of Medicine, Tokyo, Japan; ^2^ Department of Clinical Cannabinoid Research, The University of Tokyo Graduate School of Medicine, Tokyo, Japan; ^3^ Laboratory of Molecular Radiology, Center for Disease Biology and Integrative Medicine, The University of Tokyo Graduate School of Medicine, Tokyo, Japan; ^4^ Immune-Mediated Diseases Therapy Center, The University of Tokyo Graduate School of Medicine, Tokyo, Japan

**Keywords:** psoriasis, psoriatic arthritis, nailfold capillary, risk factors, tildrakizumab, regulatory T cells, elderly, prospective study

## Abstract

**Introduction:**

As a form of precision medicine, this study aimed to investigate the specific patient population that would derive the greatest benefit from tildrakizumab, as well as the mechanism of action and efficacy of tildrakizumab in reducing the occurrence of psoriatic arthritis (PsA).

**Methods:**

To achieve this, a multi-center, prospective cohort study was conducted, involving a population of 246 psoriasis patients who had not received any systemic therapy or topical finger therapy between January 2020 and April 2023. Two independent clinicians, who were blinded to the study, analyzed nailfold capillary (NFC) abnormalities, such as nailfold bleeding (NFB) and enlarged capillaries, as well as the incidence of new PsA. Additionally, the factors that determined the response of psoriasis after seven months of tildrakizumab treatment were examined. The study also examined the quantity and role of regulatory T cells (Tregs) and T helper 17 cells both pre- and post-treatment.

**Results:**

The severity of psoriasis, as measured by the Psoriasis Area and Severity Index (PASI), was found to be more pronounced in the tildrakizumab group (n=20) in comparison to the topical group (n=226). At 7 months after tildrakizumab treatment, multivariate analysis showed that those 65 years and older had a significantly better response to treatment in those achieved PASI clear or PASI 2 or less (Likelihood ratio (LR) 16.15, p<0.0001; LR 6. 16, p=0.01). Tildrakizumab improved the number and function of Tregs, which had been reduced by aging. Tildrakizumab demonstrated significant efficacy in improving various pathological factors associated with PsA. These factors include the reduction of NFB, enlargement of capillaries, and inhibition of PsA progression. The hazard ratio for progression to PsA was found to be 0.06 (95% confidence interval: 0.0007-0.46, p=0.007), indicating a substantial reduction in the risk of developing PsA.

**Discussion:**

Tildrakizumab's effectiveness in improving skin lesions can be attributed to its ability to enhance the number and function of Tregs, which are known to decline with age. Furthermore, the drug's positive impact on NFB activity and capillary enlargement, both of which are recognized as risk factors for PsA, further contribute to its inhibitory effect on PsA progression.

## Introduction

Psoriasis is a prevalent chronic inflammatory skin condition that is characterized by various treatment options (1). The field of psoriasis treatment has seen significant advancements, with the availability of at least 11 biologics specifically designed for its management (2). These treatment options encompass both oral and topical medications. The therapeutic targets for psoriasis are diverse and include the utilization of anti-tumor necrosis factor-alpha (TNF-α) antibodies, anti-interleukin (IL)-12/23p40 antibodies, and anti-IL-17 antibodies. Among such a wide variety of treatment options, treatment is often difficult because each patient responds differently to the same therapeutic agent, and what works well in one patient is often ineffective in another. Therefore, it is required to bring the most promising or most effective treatment for an individual patient. Predicting treatment response in advance and providing the most promising or most effective treatment for an individual patient is called precision medicine (3), but at present, it is difficult to predict these treatment responses in advance. In particular, among anti-IL-23 antibodies, there are more than three biologics, each of which differs in whether it is a fully human or humanized antibody, and its affinity also differs in each drug (4). Tildrakizumab, a humanized monoclonal antibody that specifically targets IL-23p19, has shown potential therapeutic effects. However, the specific patient population that would benefit from tildrakizumab remains unknown. Previous clinical trials have not provided sufficient evidence to determine the efficacy of tildrakizumab in elderly patients, likely due to the limited number of participants in this age group (5). Therefore, the objective of this study was to investigate the effectiveness of tildrakizumab in patients as part of a precision medicine approach.

In recent years, especially in Western countries and Japan, society as a whole has been aging, and the question of how to provide medical care to the elderly has become a social issue (6). Regulatory T cells (Treg) are known to be decreased in psoriasis patients, and their suppressive capacity is also known to be reduced (7). As one of the mechanisms, IL-23 is known to act on Tregs and cause pathogenic conversion to IL-17 producing cells (8). Therefore, tildrakizumab, an anti-IL-23 antibody, is expected to reverse this process. It is also known that the number of Tregs is decreased and their suppressive capacity is also decreased in the elderly (9), but there are no reports yet on an elderly patient with psoriasis.

Psoriasis is often accompanied by a range of complications that have a substantial impact on the prognosis of patients (10). One significant comorbidity associated with psoriasis is psoriatic arthritis (PsA), which results in osteoclastic arthritis and significantly diminishes patients’ quality of life (10). Dermatologists have a crucial role in diagnosing PsA as skin symptoms typically manifest years before the onset of PsA-related symptoms (10). Prompt treatment is particularly vital for PsA, as delays in treatment can lead to a decline in quality of life (11). Consequently, it is imperative to impede the progression to PsA. However, there are no reports on treatment effect of tildrakizumab for risk factors of progression to PsA, which includes NFB or enlarged capillaries (12, 13).

In recent years, biologics have been reported to inhibit the progression to PsA (14). However, the agents that have been reported primarily consist of antibodies targeting TNF-α, IL-12/23p40, and IL-17. The impact of anti-IL-23p19 antibodies remains uncertain. This study aims to examine the therapeutic response and mechanism of action of tildrakizumab, assess its influence on nailfold capillary changes, and evaluate its potential to impede the progression to PsA.

## Methods

### Patients

Data for this prospective study were collected from patients diagnosed with psoriasis vulgaris, who provided informed consent at the University of Tokyo Hospital, Takahashi clinic, or MisatoKenwa Hospital and MisatoKenwa clinic between January 2020 and April 2023. The study sample consisted of 246 patients with psoriasis vulgaris (PsV) without arthritis. PsA patients were diagnosed by rheumatologists using the CASPAR criteria (15). Only patients who had not previously received any topical treatment for distal interphalangeal (DIP) joints and nails, or any systemic treatment, were included in this study. Exclusion criteria encompassed evidence of vascular disorders, hepatitis, collagen diseases, other skin diseases, infection, and drug abuse. The nailfolds of all fingers were examined for capillaroscopic changes, and the number and distribution of nailfold videocapillaroscopy (NVC) findings in each finger were recorded. This study received approval from the University of Tokyo Ethics Board. Patients or the public were not involved in the design, conduct, reporting, or dissemination plans of this research.

### Observation of the nailfold capillaries

The nailfold capillaries were observed using the TOKU Capillaro-01 device (Toku Co., Tokyo, Japan). NVC examinations were conducted at each patient visit to determine their NVC findings. Prior to the test, patients were instructed to abstain from consuming caffeine for 12 hours. The patients were positioned in a supine position for 15 minutes at a room temperature of 22 to 25°C. Ten nailfolds were examined in each patient. The same dermatologist evaluated capillaroscopic parameters such as nailfold bleeding (NFB) and irregularly enlarged capillaries for each image. The evaluation methodology, items, and software used for the evaluation were consistent with previous descriptions (12).

### Isolation of human peripheral blood mononuclear cells and fluorescent antibody staining

Peripheral blood mononuclear cells (PBMCs) were isolated from heparinized blood samples using HetaSep (Stem Cell Technologies Inc., Vancouver, Canada) through gradient centrifugation. Following Fc blocking (I-4506, Sigma-aldrich, MO, USA), the cells were labeled with PE-labeled CD4 (MHCD0404, Thermo Fisher, Waltham, MA, USA), FITC-labeled CD25 (11-0257-42, Thermo Fisher), PE/Cy7-CD127 (25-1278-42, Thermo Fisher), APC-CD3 (17-0032-82, Thermo Fisher), PE/Cy7-CCR6 (25-1969-42, Thermo Fisher), or VioBright FITC-CXCR3 (130-106-009, Miltenyi-Biotec, Bergisch-Gladbach, Germany). The samples were then incubated at room temperature in the dark for 30 minutes. Subsequently, the samples were washed twice with PBS. After membrane staining, the cells were fixed and permeabilized using the Cytofix/Cytoperm kit (BD Biosciences). Flow cytometry analysis was performed using a BD FACSVerseTM flow cytometer with BD FACSuiteTM software (BD Biosciences, Germany). All analyses were conducted using fresh blood samples.

### T-cell proliferation and co-culture suppression assays

CD4+CD25+ regulatory T cells were isolated following the protocol provided by the manufacturer (130-091-301, Miltenyi-Biotec). The proliferative capacity of the isolated CD4+CD25+ or CD4+CD25- T cells was assessed using the protocol provided by the manufacturer (130-092-909, Miltenyi-Biotec). The suppressor capacity of T cells was investigated through co-culture assays. The proliferative capacity of isolated CD4+CD25+ or CD4+CD25- T cells (5 x 104) was analyzed by bromodeoxyuridine (BrdU), after stimulation with anti-CD3/CD28-coated beads (Invitrogen, Breda, the Netherlands) with or without exogenously added recombinant human IL-2 (12.5 U ml-1). The suppressor capacity of T cells was studied in co-culture assays. In brief, CD4+CD25- (5 x 104) T cells were stimulated with anti-CD3/CD28-coated beads in the absence and presence of decreasing numbers of CD4+CD25+ or CD4+CD25- T cells. Cell proliferation was analyzed at day 4 of the cultures by specific anti-BrdU ELISA (Roche, Meylan, France).

### Statistical analysis

To examine the factors associated with predicting the response to PASI or the development of PsA, a logistic regression model or Cox regression analysis was constructed. This analysis included PsA risk factors such as involvement of the scalp, nails, and buttocks, while adjusting for age, sex, psoriasis severity, and body mass index. Logistic regression analysis was employed to identify the key factors that coexisted with PsA, while Cox regression analysis was used to identify the key factors that predicted the development of PsA. The significance of covariate effects was determined using a two-sided Wald’s test with a p-value threshold of less than 0.05. Following the univariate analysis, the significant factors were further analyzed in a multivariate analysis. Other statistical significance was assessed using various tests, including the Mann-Whitney U-test, Wilcoxon signed-rank test, student’s t-test, paired t-test, χ2 test, Log-rank test, and Spearman’s rank correlation test. All statistical analyses were conducted using JMP Pro 14 software. A p-value of less than 0.05 was considered statistically significant.

## Results

### More severely affected patients were treated in the tildrakizumab group compared to the topical group

A total of 246 patients diagnosed with Psoriasis Vulgaris (PsV) were enrolled in the present study, as indicated in **Table 1**. Among these patients, twenty individuals received treatment with tildrakizumab, while the remaining participants were solely treated with topical agents. It was observed that patients who received tildrakizumab exhibited significantly elevated PASI scores (7.5 ± 8.6 *vs*. 2.3 ± 3.1, p < 0.0001), significantly more scalp lesions (18 (90.0%) *vs*. 109 (48.2%), p = 0.0003), and significantly more buttock lesions (18 (90.0%) *vs*. 32 (14.2%), p < 0.0001), a significantly higher proportion with NFB (19 (95.0%) *vs*. 81 (35.8%), p < 0.0001), a significantly higher proportion with enlarged capillaries (19 (95.0%) *vs*. 59 (26.1%), p < 0.0001) compared with topical group. Among the risk factors for the development of PsA (16–18), a significantly higher percentage had scalp, buttocks, NFB and enlarged capillaries. It was suggested that those at higher risk of developing PsA were more likely to be treated with tildrakizumab.

**Table 1:** Characteristics of included patients treated with tildrakizumab or topical treatments.

Baseline	Tildrakizumab (n = 20)	Topical (n = 226)	P-value
Age (years)	63 (20)	54 (20)	0.07
Sex (male/female)	15/5	147/79	0.47
BMI	23.9 (2.9)	22.9 (4.0)	0.17
Skin duration (years)	12 (11)	10 (12)	0.42
PASI	7.5 (8.6)	2.3 (3.1)	<0.0001
Scalp involvement	18 (90.0%)	109 (48.2%)	0.0003
Nail involvement	9 (45.0%)	63 (27.9%)	0.13
Buttock involvement	18(90.0%)	32 (14.2%)	<0.0001
NFB	19 (95.0%)	81 (35.8%)	<0.0001
Enlarged capillaries	19 (95.0%)	59 (26.1%)	<0.0001

Data are n (%) or mean (SD). BMI; body mass index, NFB; nailfold bleeding.

### Tildrakizumab can achieve better skin lesions in the elderly

Here, age ≥ 65 is defined as “elderly.” The number of elderly patients in the tildrakizumab group was 11. The differences in background factors between the two groups of patients are shown in **Supplementary Table S1**. There were no significant differences other than age. We first attempted to identify the clinical characteristics of the 20 patients treated with tildrakizumab who achieved PASI clear or PASI ≤ 2 at 7 months post-treatment (**Table 2**). In logistic regression analysis, we compared age, gender, BMI, duration of skin lesions, PASI, scalp, nail, and buttock lesions, NFB, and presence of enlarged capillaries. As a result, age≥65 was the only significant variable in the multivariate analysis. Similarly, we identified clinical characteristics of patients who were able to achieve PASI ≤ 2 (**Table 3**). Similarly, age≥65 was the only significant variable. In summary, the results suggest that age is an important factor in predicting treatment responsiveness of skin lesions after 7 months of tildrakizumab.

**Table 2:** Factors that predict PASI clear after 7 months of tildrakizumab treatment.

	Univariate		Multivariate	
	Likelihood ratio	p-value	Likelihood ratio	p-value
Age ≧ 65 (years)	17.09	<0.0001	16.15	<0.0001
Sex (male)	0.61	0.44	0.76	0.38
BMI ≧25	0.31	0.58		
Skin duration ≧ 10 (years)	0.91	0.34		
PASI ≧10	5.60	0.02	3.26	0.07
Scalp involvement	2.57	0.11		
Nail involvement	0.74	0.39		
Buttock involvement	2.57	0.11		
NFB	1.24	0.27		
Enlarged capillaries	1.24	0.27		

CI, confidence interval; BMI, body mass index; PASI, psoriasis area and severity index; NFB, nailfold bleeding.

**Table 3:** Factors that predict PASI ≦ 2 after 7 months of tildrakizumab treatment.

	Univariate		Multivariate	
	Likelihood ratio	p-value	Likelihood ratio	p-value
Age ≧ 65 (years)	7.64	0.006	6.16	0.01
Sex (male)	0.00	1.00	0.37	0.54
BMI ≧25	0.21	0.65		
Skin duration ≧ 10 (years)	1.86	0.17		
PASI ≧10	2.41	0.12	1.24	0.26
Scalp involvement	0.95	0.33		
Nail involvement	0.05	0.82		
Buttock involvement	0.95	0.33		
NFB	0.46	0.50		
Enlarged capillaries	0.46	0.50		

CI, confidence interval; BMI, body mass index; PASI, psoriasis area and severity index; NFB, nailfold bleeding.

### Tildrakizumab improves the number and function of Tregs reduced by aging

**Figure 1:**
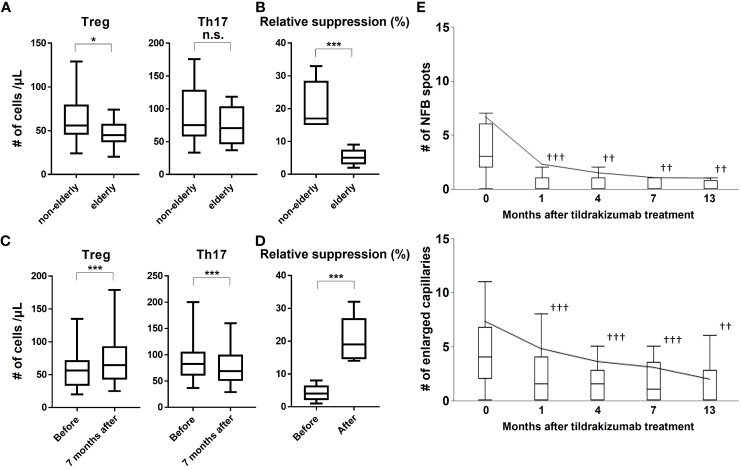
Treg number and function are reduced in the elderly, and tildrakizumab improves them and also NFB and enlarged capillaries, risk factors for progression to PsA. We examined the numbers of Treg and Th17 in the peripheral blood of elderly and non-elderly subjects **(A)** and their suppressive ability **(B)**. We also examined the change in the number of Tregs and Th17 **(C)** and their suppressive capacity before and after Tildrakizumab treatment **(D)**. The number of NFBs and enlarged capillaries were examined before and at 1, 4, 7, and 13 months after treatment with tildrakizumab **(E)**. Box plot shows median, 25th, and 75th percentile, and whiskers show the standard deviation. *p<0.05, ***p<0.005. ††p<0.01, †††p<0.005 *vs*. before treatment. n.s. = not significant.

The above analysis suggests that age is an important factor in determining response to treatment with tildrakizumab. We focused on Treg as one factor explaining this phenomenon. Since it has been reported that the number of Tregs is decreased and Treg function is impaired in psoriasis and in the elderly (9), we first examined differences in the number of Tregs and T helper 17 (Th17) cells with age in psoriasis patients (**Figure 1A**). The number of Treg was significantly decreased in the elderly, whereas that of Th17 showed no age-related differences (**Figure 1A**). Analysis of the reduced Treg function also revealed that the suppressive capacity of Tregs was also reduced (**Figure 1B**). Therefore, we examined these Tregs and Th17 before and after treatment with tildrakizumab (**Figure 1C**). Tregs were significantly increased and Th17 was significantly decreased after treatment with Tildrakizumab (**Figure 1C**). The suppressive function of Treg was also improved by treatment with tildrakizumab (**Figure 1D**). These findings suggest that one factor explaining the higher efficacy of tildrakizumab in the elderly may be its ability to improve the number and function of reduced Tregs.

### Tildrakizumab improves NFB and enlarged capillaries, risk factors for progression to PsA, and inhibits the progression to PsA

Thus, tildrakizumab acts at the cellular level and improves the skin lesions of psoriasis. Th17 cells produce inflammatory cytokines, which have also been implicated in nailfold capillary abnormalities (12). In fact, it was discovered that treatment with tildrakizumab resulted in significant improvements in NFB and enlarged capillaries, both of which are risk factors for the development of PsA. These improvements were observed as early as one month after treatment initiation and were sustained throughout the study period (**Figure 1E**). During the course of the study, 36 patients in the topical group and 1 patient in the tildrakizumab group developed PsA. A multivariate analysis using a Cox proportional hazards model identified age, nail lesions, NFB, and enlarged capillaries as risk factors for the progression to PsA. However, treatment with tildrakizumab was found to be effective in preventing the progression to PsA (**Table 4**). In conclusion, these findings demonstrate that tildrakizumab not only alleviates psoriasis at the cellular level but also improves NFB and enlarged capillaries, which are known risk factors for the development of PsA, ultimately inhibiting the progression to PsA.

**Table 4:** Factors that predict or prevent the development of PsA.

	Univariate			Multivariate with NFB			Multivariate with enlarged capillaries		
	Hazard ratio	95% CI	p-value	Hazard ratio	95% CI	p-value	Hazard ratio	95% CI	p-value
Age (/1year)	1.02	1.00-1.04	0.06	1.02	1.00-1.04	0.02	1.02	1.00-1.04	0.02
Sex (Female)	0.99	0.49-1.97	0.97	1.51	0.73-3.13	0.26	1.36	0.67-2.75	0.39
BMI									
≧25 *vs* 25>	1.00	0.50-2.03	0.99						
PASI									
≧10 *vs* 10>	0.79	0.19-3.31	0.75						
Scalp involvement	1.28	0.66-2.48	0.47						
Nail involvement	2.85	1.49-5.46	0.002	3.10	1.60-6.02	0.0008	2.44	1.23-4.82	0.01
Buttock involvement	1.01	0.47-2.20	0.97						
NFB	2.15	1.11-4.15	0.023	2.92	1.44-5.93	0.003			
Enlarged capillaries	3.44	1.78-6.66	0.0002				4.61	2.27-9.38	<0.0001
Tildrakizumab	0.17	0.02-1.28	0.09	0.06	0.007-0.46	0.007	0.04	0.01-0.34	0.003

CI, confidence interval; BMI, body mass index; PASI, psoriasis area and severity index; NFB, nailfold bleeding.

## Discussion

In this study, a significantly higher percentage of patients in the tildrakizumab group had scalp, buttocks, NFB and enlarged capillaries, and those at higher risk of progressing to PsA were treated with more tildrakizumab compared to the topical group (**Table 1**). Among those treated with tildrakizumab, those who achieved better skin lesions at 7 months post-treatment were older (**Tables 2** and **3**). One factor that may explain this is the importance of Tregs, which were found to be more reduced and less functional in elderly patients with psoriasis (**Figures 1A, B**). These abnormalities improved with treatment with tildrakizumab (**Figures 1C, D**). One factor that may explain the higher efficacy of tildrakizumab in elderly patients is that it may be more effective in improving the number and function of reduced Tregs. Tildrakizumab also improved NFB and enlarged capillaries, one of the risk factors for progression to PsA (**Figure 1E**), and inhibited progression to PsA (**Table 4**). These results suggest that tildrakizumab improved psoriasis at the cellular level, ameliorated NFB and enlarged capillaries, one of the risk factors for progression to PsA, and inhibited progression to PsA.

To date, factors determining response to treatment with tildrakizumab have not been identified. For the first time in this study, tildrakizumab restores the number and function of Tregs and is more effective, especially in the elderly. It has been reported that Treg function is reduced in the elderly (9). Some researchers have reported a decreased percentage of Tregs in peripheral blood of psoriasis patients (19–21), while others showed no difference in circulating Treg frequency (22–25). However, differences in Tregs by age have not been investigated in patients with psoriasis. This study suggested that Tregs may be reduced in number and function especially in the elderly psoriasis patients. Tildrakizumab, an anti-IL-23 antibody that targets IL-23, which is known to act on Tregs, as a therapeutic target, was thought to be more effective in the elderly by targeting them.

PsA is a disease in which immune abnormalities are strongly implicated; it has been suggested that in the development to PsA, the disease progresses to skin lesions, immune abnormalities, subclinical and clinical PsA (26). Tildrakizumab, an anti-IL-23 antibody, acts at the cellular level and is thought to improve the skin lesions of psoriasis by inhibiting the differentiation and proliferation of Th17 cells and the pathogenic conversion of Tregs to Th17 cells (27). IL-23 produced by macrophages, dendritic cells, and B cells (28) can act on Tregs, making them Th17-like and exacerbating the skin lesions of psoriasis (27). Treg functions include reducing inflammation and autoimmunity. In recent years, a number of reports have emerged that the use of biologics for skin lesions can inhibit their progression to PsA (14). However, only the types of antibodies were reported, such as anti-TNF-α, anti-IL-12/23p40, anti-IL-17, and anti-IL-23p19 antibodies, and there are few reports on whether individual antibodies inhibit progression to PsA. This study shows for the first time that tildrakizumab improves NFB and enlarged capillaries, which are risk factors for PsA, and reduces progression to PsA.

Capillary abnormalities such as NFB and enlarged capillaries are thought to be secondary to the systemic inflammation of psoriasis (12). Indeed, the correlation between serum cytokine levels and capillary abnormalities may support these views. Therefore, it is reasonable that tildrakizumab, which targets IL-23, an inflammatory cytokine, ameliorated nailfold capillary abnormalities. A strong association between inflammatory cytokines and capillary abnormalities is suggested by the reported efficacy of brodalumab, an anti-IL-17RA antibody, in the treatment of systemic sclerosis, one of the most common diseases showing nailfold capillary abnormalities (29–31).

Taken together, tildrakizumab is particularly effective in the elderly. Its high safety profile also makes it safe for use on the elderly (32). The mechanism of action was suggested to be targeting Tregs. Improvement of risk factors for progression to PsA and, indeed, inhibition of progression to PsA were revealed. The primary drawback of this study is its exclusive focus on Japanese patients. The potential influence of genetic variations between the Japanese population and other ethnic groups on treatment response cannot be overlooked. Another limitation of this study is the low patient number. To ascertain the efficacy of tildrakizumab in preventing PsA in psoriasis, it is anticipated that future large-scale clinical trials will encompass diverse ethnic populations.

## Data availability statement

The original contributions presented in the study are included in the article/**Supplementary Material**. Further inquiries can be directed to the corresponding author.

## Ethics statement

The studies involving humans were approved by the ethics committee of the University of Tokyo Graduate School of Medicine. The studies were conducted in accordance with the local legislation and institutional requirements. The participants provided their written informed consent to participate in this study.

## Author contributions

TF: Conceptualization, Data curation, Formal Analysis, Investigation, Methodology, Resources, Software, Validation, Visualization, Writing – original draft, Writing – review & editing. TY: Data curation, Formal Analysis, Investigation, Methodology, Resources, Software, Validation, Visualization, Writing – original draft, Writing – review & editing. AE: Data curation, Formal Analysis, Investigation, Methodology, Resources, Software, Validation, Visualization, Writing – original draft, Writing – review & editing. YN: Data curation, Formal Analysis, Investigation, Methodology, Resources, Software, Validation, Visualization, Writing – original draft, Writing – review & editing. StT: Data curation, Formal Analysis, Investigation, Methodology, Resources, Software, Validation, Visualization, Writing – original draft, Writing – review & editing. AY: Data curation, Formal Analysis, Investigation, Methodology, Resources, Software, Validation, Visualization, Writing – original draft, Writing – review & editing. SkT: Data curation, Formal Analysis, Investigation, Methodology, Resources, Software, Validation, Visualization, Writing – original draft, Writing – review & editing. HK: Data curation, Formal Analysis, Investigation, Methodology, Resources, Software, Validation, Visualization, Writing – original draft, Writing – review & editing. KM: Data curation, Formal Analysis, Investigation, Methodology, Resources, Software, Validation, Visualization, Writing – original draft, Writing – review & editing. SS: Supervision, Validation, Visualization, Writing – original draft, Writing – review & editing, Conceptualization, Data curation, Formal Analysis, Funding acquisition, Investigation, Methodology, Project administration, Resources, Software. AY: Data curation, Formal Analysis, Funding acquisition, Investigation, Methodology, Project administration, Resources, Software, Supervision, Validation, Visualization, Writing – original draft, Writing – review & editing.
