# Supplementary material for: The optimal use of tildrakizumab in the elderly via improvement of Treg function and its preventive effect of psoriatic arthritis
Source: Front Immunol. 2023 Oct 19;14:1286251. doi: 10.3389/fimmu.2023.1286251 (PMC10620742; doi:10.3389/fimmu.2023.1286251)
Supplement: Supplementary file 1 [file Table_1.docx]

**The optimal use of tildrakizumab in the elderly via improvement of Treg function and its preventive effect of psoriatic arthritis.**

Takemichi Fukasawa^1, 2^, Takashi Yamashita^1^, Atsushi Enomoto^3^, Yuta Norimatsu^1^, Satoshi Toyama^1^, Asako Yoshizaki-Ogawa^1^, Shoko Tateishi^4^, Hiroko Kanda^4^, Kiyoshi Miyagawa^3^, Shinichi Sato^1^, and Ayumi Yoshizaki^1, 2, 4^*

^1^ Department of Dermatology, Psoriasis center, The University of Tokyo Graduate School of Medicine, Tokyo, Japan.

^2^ Department of Clinical Cannabinoid Research, The University of Tokyo Graduate School of Medicine

^3^ Laboratory of Molecular Radiology, Center for Disease Biology and Integrative Medicine, The University of Tokyo Graduate School of Medicine, Tokyo, Japan.

^4^ Immune-Mediated Diseases Therapy Center, The University of Tokyo Graduate School of Medicine, Tokyo, Japan.

**Correspondence and reprint requests to:**

Ayumi Yoshizaki, M.D., Ph.D., Department of Dermatology, Psoriasis center, and Department of Clinical Cannabinoid Research, The University of Tokyo Graduate School of Medicine, 7-3-1 Hongo, Bunkyo-ku, Tokyo 113-8655, Japan.

Telephone: +81-3-5800-8661, Fax: +81-3-3814-1503

E-mail: [ayuyoshi@me.com](mailto:ayuyoshi@me.com)

**Supplementary Tables**

**Supplementary Table S1. Characteristics of elderly and non-elderly patients treated with tildrakizumab.**

| Baseline | Elderly  (n = 11) | Non-elderly  (n = 9) | P-value |
| --- | --- | --- | --- |
| Age (years) | 77 (5) | 44 (15) | 0.0002 |
| Sex (male/female) | 8/3 | 7/2 | 1.00 |
| BMI | 23.5 (2.6) | 24.4 (3.2) | 0.62 |
| Skin duration (years) | 14 (12) | 9 (9) | 0.38 |
| PASI | 4.2 (2.9) | 11.6 (11.4) | 0.22 |
| Scalp involvement | 10 (90.9%) | 8 (88.9%) | 1.00 |
| Nail involvement | 6 (54.5%) | 3 (33.3%) | 0.41 |
| Buttock involvement | 10 (90.9%) | 8 (88.9%) | 1.00 |
| NFB | 10 (90.9%) | 9 (100.0%) | 1.00 |
| Enlarged capillaries | 10 (90.9%) | 9 (100.0%) | 1.00 |

Data are n (%) or mean (SD). BMI; body mass index, NFB; nailfold bleeding.
